# Mapping anhedonia-specific dysfunction in a transdiagnostic approach: an ALE meta-analysis

**DOI:** 10.1007/s11682-015-9457-6

**Published:** 2015-10-20

**Authors:** Bei Zhang, Pan Lin, Huqing Shi, Dost Öngür, Randy P. Auerbach, Xiaosheng Wang, Shuqiao Yao, Xiang Wang

**Affiliations:** 1 Medical Psychological Institute, The Second Xiangya Hospital of Central South University, 139 Renmin (M) Road, Changsha, Hunan 410011 China; 2 Key Laboratory of Biomedical Information Engineering of Education Ministry, Institute of Biomedical Engineering, Xi’an Jiaotong University, Xi’an, Shanxi 710049 China; 3 Department of Psychology, Shanghai Normal University, Shanghai, 200234 China; 4 Harvard Medical School and McLean Hospital, 115 Mill Street, Belmont, MA 02478 USA; 5 Department of Anatomy and Neurobiology, Xiangya School of Medicine, Central South University, Changsha, 410013 China

**Keywords:** Anhedonia, Activation likelihood estimation (ALE), Meta-analysis, Transdiagnostic, Major depressive disorder, Schizophrenia

## Abstract

Anhedonia is a prominent symptom in neuropsychiatric disorders, most markedly in major depressive disorder (MDD) and schizophrenia (SZ). Emerging evidence indicates an overlap in the neural substrates of anhedonia between MDD and SZ, which supported a transdiagnostic approach. Therefore, we used activation likelihood estimation (ALE) meta-analysis of functional magnetic resonance imaging studies in MDD and SZ to examine the neural bases of three subdomains of anhedonia: consummatory anhedonia, anticipatory anhedonia and emotional processing. ALE analysis focused specifically on MDD or SZ was used later to dissociate specific anhedonia-related neurobiological impairments from potential disease general impairments. ALE results revealed that consummatory anhedonia was associated with decreased activation in ventral basal ganglia areas, while anticipatory anhedonia was associated with more substrates in frontal-striatal networks except the ventral striatum, which included the dorsal anterior cingulate, middle frontal gyrus and medial frontal gyrus. MDD and SZ patients showed similar neurobiological impairments in anticipatory and consummatory anhedonia, but differences in the emotional experience task, which may also involve affective/mood general processing. These results support that anhedonia is characterized by alterations in reward processing and relies on frontal-striatal brain circuitry. The transdiagnostic approach is a promising way to reveal the overall neurobiological framework that contributes to anhedonia and could help to improve targeted treatment strategies.

**Electronic supplementary material:**

The online version of this article (doi:10.1007/s11682-015-9457-6) contains supplementary material, which is available to authorized users.

## Introduction

Anhedonia is defined as ‘markedly diminished interest or pleasure in all, or almost all, activities most of the day, nearly every day’ (Association and Association 1994). Although anhedonia has long been considered a prominent symptom in neuropsychiatric disorders, especially major depressive disorder (MDD) and schizophrenia (SZ) (Association and Association 1994), its underlying neurobiological mechanisms remain poorly understood. Recently, evidence has emerged to indicate overlap of behavioral, cognitive processing and neurobiological abnormalities between MDD and SZ patients with marked clinical anhedonia (Gradin et al. 2011; Whitton et al. 2015). As a result of this finding and the advocacy of the NIMH Research Domain Criteria (RDoC) project (Insel et al. 2010), investigators are seeking to use the basic behavioral dimension of functioning, rather than traditional diagnostic categories, to identify transdiagnostic neural markers of anhedonia (Corral-Frias et al. 2015; Cuthbert and Insel 2013; Markou et al. 2009). A quantitative meta-analysis of pooled neuroimaging studies of anhedonia in MDD and SZ, discussed within the RDoC framework, is a promising approach to investigate the neural substrates of anhedonia. However, it is important to be mindful of the complexity and multi-faceted nature of clinical anhedonia to understand neuroimaging studies in this field.

Based on the latest studies, anhedonia is a multidimensional construct and should not simply be considered as ‘loss of an ability to experience pleasure’. Deficits in other reward processes, such as valuation, motivation and decision-making, may lead to behaviors that can be interpreted as anhedonia (Der-Avakian and Markou 2012; Gold et al. 2008; Leventhal et al. 2006). Hence, distinguishing the deficits in different cognitive subcomponents of anhedonia is essential to identify its neurobiological substrates. Some investigators have suggested dichotomizing anhedonia into ‘consummatory anhedonia’ (the hedonic response to rewards) and ‘anticipatory anhedonia’ (diminished motivation to pursue rewards) (Treadway and Zald 2011). Others hold that studies should move away from conceptualization of anhedonia as a steady-state, mood-like phenomena, and instead focus on the reward-related motivational/decisional-making aspect (Treadway et al. 2012; Whitton et al. 2015). Still others propose to bridge the gap between preclinical and clinical studies by isolating the neural substrates of various processes, such as sensing a pleasant stimulus or anticipating expected rewards, computing value and associated costs, determining effort required, deciding to obtain the reward, increasing motivation and performing the action (Der-Avakian and Markou 2012). Changing research strategies and models demand more complicated and elaborate experimental designs to investigate the underlying neural bases of anhedonia.

Several experimental paradigms have been developed in last decade to explore the specific processes of anhedonia beyond hedonic capacity, such as anticipation, motivation and reinforcement learning. However, only a few task-based functional magnetic resonance imaging (fMRI) studies have separated anhedonia deficits into different processing stages. Early neuroimaging studies frequently adopted the passive picture-viewing task based on the viewpoint that anhedonia represents a diminished responsiveness to positive-valence stimuli (Hooker et al. 2014; Mitterschiffthaler et al. 2003; Shi et al. 2013). More recently, some experimental reward paradigms, especially the monetary incentive delay task (MID) (Admon et al. 2015; Balodis and Potenza 2015; Elman et al. 2009), have been increasingly used to detect neural substrates of anhedonia. Those studies have typically explored brain activity impairments in experience/consummatory and motivational/anticipatory stages separately, and they discussed the link between anhedonia and the reward-processing components of ‘liking’ and ‘wanting’ from the preclinical literature (Berridge and Robinson 2003; Dillon et al. 2008). Specifically, the consummatory stage was primarily associated with dysfunctions of reward coding and evaluation, and the anticipatory stage was associated with advanced cognitive functions, such as motivation and decision-making. Recently, although other paradigms have been developed to assess a specific aspect of anhedonia-related processing, such as the Effort Expenditure for Rewards Task (EEfRT), which focuses on motivation and effort-based decision-making (Fervaha et al. 2013; Treadway et al. 2009; Yang et al. 2014) and the Probabilistic Learning Task (PRT), which focuses on reward learning (Pizzagalli et al. 2008), no neuroimaging studies have used these two paradigms in MDD or SZ patients. In fact, to date the majority of studies have explored the neurobiology of the three subdomains of anhedonia, which involve different neurocognitive process and functional neuroanatomy bases: consummatory anhedonia, anticipatory anhedonia and emotional (positive stimuli) processing (Berridge and Robinson 2003; Dillon et al. 2008; Smoski et al. 2011).

Neuroimaging studies of the three subdomains of anhedonia in MDD and SZ patients have reached inconsistent and even contradictory findings. For instance, several studies that used a reward-related task, most often MID, indicated ventral striatal blunting in depressed adults (Knutson et al. 2008; Kumar et al. 2008; Ubl et al. 2015) and even in never-depressed youth with family history of MDD (Gotlib et al. 2010). Another study using the same experimental paradigm (MID) showed no significant group difference in ventral striatum between unmedicated MDD and controls after correction (Pizzagalli et al. 2009). Furthermore, Pizzagalli et al. (2008) reported decreased activation in dorsal anterior cingulate (dACC) during the MID task in MDD patients compared with healthy controls, whereas other studies found increased activation of dACC in MDD (Gorka et al. 2014; Knutson et al. 2008). Similar contradictory results have been observed in SZ patients, especially in the experience/consummatory stage. When presented with reward stimuli, SZ patients showed impairments in both ventral striatal and prefrontal areas (da Silva Alves et al. 2013; Walter et al. 2009a), impairments in prefrontal cortex but not basal ganglia areas (Waltz et al. 2010) or no impairments in either area (Mucci et al. 2015; Simon et al. 2010). These inconsistencies among studies may reflect differences in sample size, sample characteristics (average age, sexual ratio, medicated or not), paradigms used, and fMRI analysis methods.

The development of meta-analyses methods for neuroimaging data provide a valuable tool for combining data across diverse studies and building consensus in identification of neuroanatomical correlates of specific behavior. To date, the main methods used for the meta-analyses of neuroimaging data could be divided into two categories: the regions of interest (ROI) based meta-analytic methods, and the voxel-based meta-analytic methods. The ROI-based methods allow for optimal statistical analyses but are based on a priori hypotheses therefore being affected by a limited and potentially biased inclusion of brain regions (Rotge et al. 2009; Radua and Mataix-Cols 2012). On the other hand, voxel-based methods have a more exhaustive and unbiased inclusion of studies but have some statistically limitation. Activation likelihood estimation (ALE), proposed by Turkeltaub and Laird (Turkeltaub et al. 2002; Laird et al. 2005), is probably the most common algorithm for voxel-based meta-analyses.

Besides the clear mathematical logic and operation steps, ALE method has several advantages over ROI-based meta-analyses through inputting the foci of activation instead of labels, weighting the foci by the number of participants in each study, yielding a quantitative estimate of the probability of activation, identifying common activations across different studies (Laird et al. 2005; Wager et al. 2007; Eickhoff et al. 2009; Eickhoff et al. 2012). More importantly, the recent change from fixed-effects to random-effects inference in analyses (Eickhoff et al. 2009) and the revision for multiple comparison correction (Eickhoff et al. 2012) made ALE to become a more reliable statistic meta-analysis approach.

Therefore, the ALE meta-analysis method was used in this review to make an objective, systematic, and quantitative analysis of the previous literature and synthesize the anhedonia-related, task-based fMRI findings in MDD and SZ from three subdomains of anhedonia: consummatory anhedonia, anticipatory anhedonia, and emotional processing. Specifically, we first performed the within-group ALE meta-analyses to provide a context for interpreting the anhedonia-related between-group differences. In the between-group analysis, the transdiagnostic ALE analysis of the literature on MDD and SZ was examined to provide an integrated framework of the neural bases of anhedonia, and the analysis focused specifically on MDD or SZ was used later to dissociate specific anhedonia-related neurobiological impairments from potential disease general impairments. The transdiagnostic approach provides the opportunity to identify an overall neurobiological framework for a specific symptom or behavior across multiple disease states and has the potential to improve targeted treatment strategies.

## Methods

### Data sources

Studies focused on the neurobiology of anhedonia in patients with MDD or SZ were identified through computerized literature searches using PubMed and Web of Science. We reviewed all papers published in the English language up to August 1, 2015. The key words used for the search were ‘anhedonia’, ‘hedonic’, ‘emotional withdrawal’, ‘pleasure deficit’, ‘apathetical social withdrawal’, ‘functional magnetic resonance’ and ‘fMRI’. A total of 571 English publications were initially identified using this process. Another three articles were obtained from reference lists of prior reviews (Whitton et al. 2015; Zhang et al. 2013). In summary, these search procedures yielded an initial pool of 574 potential articles for inclusion.

In prior neuroimaging studies focused on anhedonia, the most common paradigms adopted were the MID, reinforcement learning task (RLT) and emotional experience task (EET). For MID, RLT or some modified reward tasks, subjects initially see a cue indicating that they will have an opportunity to obtain a certain amount of reward (e.g., money), then they perform a task, and they receive immediate feedback (by obtaining or not obtaining the reward). The process between the cue and the task refers to anticipation stage, and the in-the-moment experience during receipt of a reward refers to consummatory stage. Besides, the emotional experience task (EET) is associated with a passive viewing of positive-valence stimuli that requires no response. Therefore, based on different cognitive process, we divided anhedonia into three subdomains: anticipatory anhedonia, consummatory anhedonia, and emotional processing.

### Inclusion criteria

**Fig. 1:**
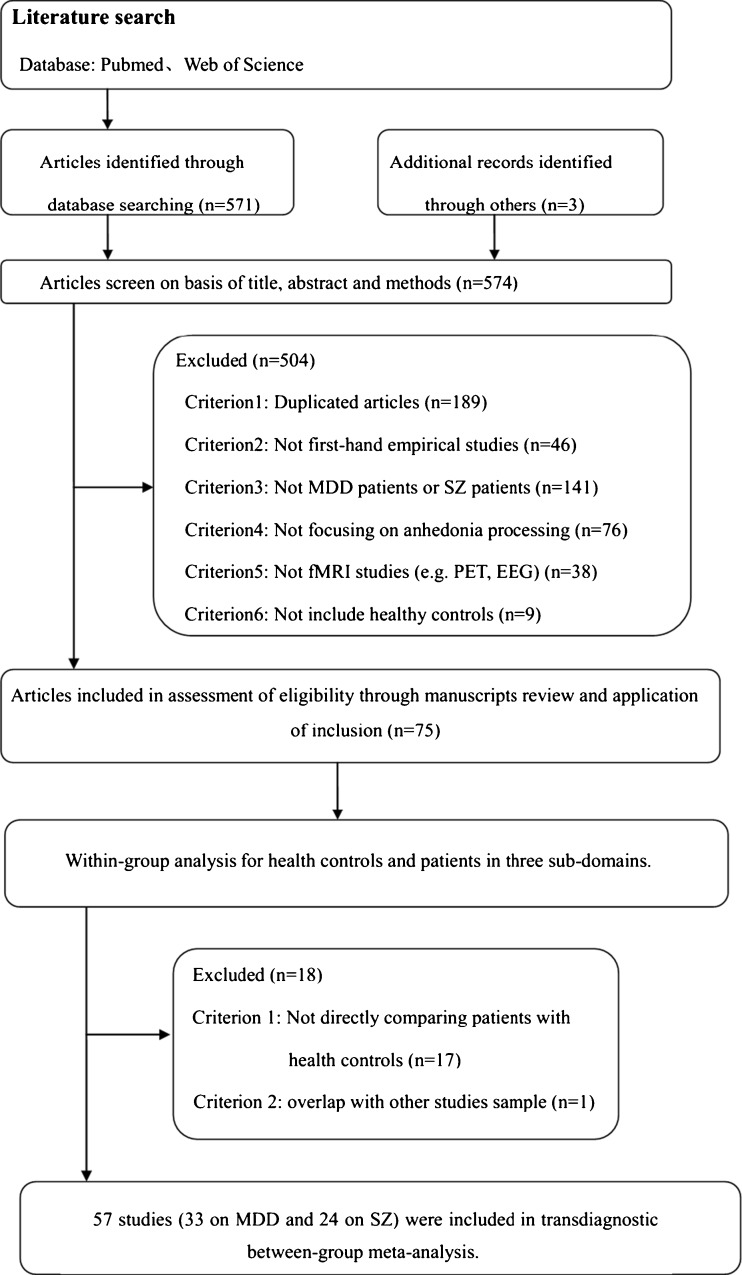
Flowchart describing the process of study identification and selection

For the current meta-analysis, once duplicate articles were removed, the following eight inclusion criteria were utilized: (1) first-hand empirical studies; (2) subjects were healthy controls or patients with either MDD or SZ; (3) MDD or SZ were diagnosed according to DSM-IV(−TR) or ICD-10; (4) studies focused on anhedonia though task-related paradigms; (5) studies examined neuronal activity related to anhedonia using fMRI; (6) studies identified foci of task-related neural changes in active conditions (e.g., emotional picture or monetary gain) and/or contrasted an active and a control condition (neutral picture or fixation cross); (7) coordinates were reported in either standard Talairach space or Montreal Neurologic Institute (MNI) space. Figure 1 shows the process of study identification and selection. From the identification and selection of studies, coordinate results of within-group activations and between-group differences were divided into 4 groups: activations in patients, activations in healthy controls, increases in patients relative to controls, and increases in controls relative to patients.

### Activation likelihood estimation (ALE) procedure

Meta-analysis was performed using the ALE software implemented in GingerALE version 2.3.2 (http://brainmap.org). This ALE version used a random effect model and weighted for sample size of the original studies (Laird et al. 2005). In ALE, activation foci reported in original studies are treated as 3D Gaussian distributions centered at the reported coordinates. Activation probabilities are then calculated for each standard-space voxel to construct ALE maps for contrasts of interest. To determine the reliability of the ALE map, null-distributions are generated by analyzing the distribution of ALE values across independent studies, which is conceptually similar to using permutation tests of individual voxels across experiments. The observed values in the ALE distribution are then compared to the null distribution in order to assign probability estimates to the observed (experimental) data.

For the present meta-analysis, single studies were used respectively to perform meta-analyses of anhedonia for each of the three subdomains. Coordinates of the foci of activation reported in MNI were transformed to Talairach space using the icbm2tal in GingerALE. The threshold of statistical significance was set at *p* < 0.01 and corrected for multiple testing using the false discovery rate (FDR) with a minimum cluster size of 400 mm^3^. For visualization, whole-brain maps of thresholded ALE maps were imported into multi-image analysis GUI (MANGO; http://ric.uthscsa.edu/mango) and overlaid onto a standardized anatomical template in Talairach space (www.brainmap.org/ale/colin1.1.nii).

## Results

### Article inclusion

Figure 1 shows the study selection flowchart, and Table 1 shows the pooled data for all contrasts. A total of 32 articles with 684 healthy controls reporting 353 foci, and 28 articles with 562 patients reporting 214 foci were included in within-group analysis. A total of 57 articles (33 articles on MDD and 24 on SZ) with 986 patients and 1041 healthy controls and reporting 453 foci were included in the transdiagnostic meta-analysis. There were no significant differences in age or sex between patients (34.33 ± 8.55 years old; 51.09 % men) and healthy controls (32.12 ± 7.09 years old; 51.71 % men), MDD patients (36.40 ± 8.79 years old; 40.82 % men) and healthy controls (33.85 ± 8.42 years old; 39.80 % men) or between SZ patients (30.75 ± 6.96 years old; 63.64 % men) and healthy controls (31.85 ± 3.70 years old; 71.33 % men).

**Table 1:** Published fMRI studies included in the between-group ALE analysis of anhedonia across MDD and SZ

Author, year	Contrasts	Healthy controls		Patients		Medication (Y/N)	Paradigm	Experimental condition	Stereotactic space
		Age (years)	N (f/m)	Age (years)	N (f/m)				
Ubl et al. 2015	MDD vs. CON	43.96	28 (15/13)	46.00	30 (16/14)	N	MID	High reward vs. control condition	MNI
Gorka et al. 2014	CON, MDD vs. CON	29.50	18 (13/5)	25.40	9 (6/3)	Y(11.1 %)	Reward anticipation task	Reward vs. no incentive	MNI
Hall et al. 2014	MDD, CON, MDD vs. CON	37.38	29 (16/13)	37.02	29 (16/13)	Y (100 %)	RLT	Reward acquisition	TAL
Fournier et al. 2013	MDD vs. CON	32.60	28 (16/12)	30.60	26 (18/8)	Y (69.2 %)	EET	Happy faces	TAL
Stuhrmann et al. 2013	MDD, CON, MDD vs. CON	40.30	35 (21/14)	40.10	35 (21/14)	Y (100 %)	EET	Happy vs. neutral faces	MNI
Stoy et al. 2012	MDD, CON, MDD vs. CON	39.50	15 (5/10)	41.90	15 (5/10)	N	MID	Gain vs. neutral	TAL
Robinson et al. 2012	MDD vs. CON	31.00	14 (6/8)	36.00	13 (5/8)	N	RLT	Unexpected reward	MNI
Dichter et al. 2012	MDD vs. CON	27.90	19 (12/7)	24.50	19 (15/4)	Y (26.3 %)	MID	Reward vs. no-reward	MNI
Chantiluke et al. 2012	MDD vs. CON	16.30	21 (11/10)	16.20	20 (10/10)	N	RLT	Rewarded vs. non-rewarded target	TAL
Derntl et al. 2011	MDD vs. CON	32.90	15 (9/6)	34.10	15 (9/6)	Y (86.7 %)	EET	Happy condition	MNI
Smoski et al. 2011	MDD vs. CON	26.20	13	34.40	9	Y (44.4 %)	MID	Reward (or positive) vs. neutral codition	MNI
Gradin et al. 2011	MDD, SZ, CON, MDD vs. CON, SZ vs. CON	40.64	17 (7/10)	45.27	15 (6/9)	Y (100 %)	RLT	Us(water) vs. cs	MNI
Bermpohl et al. 2009	CON, MDD vs. CON	24.10	21 (10/11)	43.40	15 (12/3)	Y (93.3 %)	EET	Cued vs. uncued emotional pictures; Emotional > neutral expectancy	MNI
Walter et al. 2009b	MDD vs. CON	34.60	24 (18/6)	40.00	19 (11/8)	N	EET	Emotional pictures vs. at rest	MNI
Remijnse et al. 2009	MDD vs. CON	32.00	27 (19/ 8)	34.00	20 (8/12)	N	RLT	Reward outcome vs. baseline	MNI
McCabe et al. 2009	MDD vs. CON	28.50	14 (9/5)	27.80	13 (11/3)	N	EET	Pleasant stimulates	MNI
Pizzagalli et al. 2009	MDD vs. CON	38.80	31 (13/18)	43.17	30 (15/15)	N	MID	Reward vs. no-reward	MNI
Smoski et al. 2009	MDD vs. CON	30.80	15 (9/6)	34.80	16 (9/7)	N	WOF	Reward vs. no-reward	TAL
Osuch et al. 2009	MDD vs. CON	23.50	15 (11/4)	22.60	16 (11/5)	Y (18.6 %)	EET	Favorite vs. neutral music	TAL
Forbes et al. 2009	MDD, CON, MDD vs. CON	13.10	28 (21/7)	13.50	15 (10/5)	N	RLT	Reward vs. no-reward	TAL
Knutson et al. 2008	MDD, CON, MDD vs. CON	28.67	12 (4/8)	30.71	14 (5/9)	Y (100 %)	MID	Reward vs. no-reward	TAL
Kumar et al. 2008	MDD, CON, MDD vs. CON	42.00	18 (11/7)	45.30	15 (9/6)	Y (100 %)	RLT	US(water) vs. CS	MNI
Steele et al. 2007	MDD, CON, MDD vs. CON	43.00	14 (7/7)	45.90	15 (11/4)	Y (93.3 %)	Gambling task	Win feedback	MNI
Fu et al. 2007	MDD vs. CON	42.80	19 (11/8)	43.20	19 (13/6)	N	EET	Happy faces	TAL
Epstein et al. 2006	MDD, CON, MDD vs. CON	32.00	12 (7/5)	35.60	10 (9/1)	N	EET	Positive words	TAL
Keedwell et al. 2005	MDD, CON, MDD vs. CON	36.00	12 (8/4)	43.00	12 (8/4)	Y (91.7 %)	EET	Happy vs. neutral stimuli	TAL
Gotlib et al. 2005	MDD vs. CON	30.80	18 (13/5)	35.20	18 (13/5)	Y (50.0 %)	EET	Happy vs. neutral faces	TAL
Tremblay et al. 2005	MDD vs. CON	29.33	12 (7/5)	34.83	12(6/6)	Y (100 %)	EET	On condition vs. off condition	TAL
Surguladze et al. 2005	MDD vs. CON	35.10	14 (6/8)	42.30	16 (6/10)	Y (56.3 %)	EET	Happy facial expressions	TAL
Canli et al. 2004	MDD vs. CON	30.70	15 (12/3)	35.10	15 (12/3)	Y (46.7 %)	EET	Happy vs. neutral words	TAL
Mitterschiffthaler et al. 2003	MDD, CON, MDD vs. CON	48.30	7 (7/0)	46.30	7 (7/0)	Y (100 %)	EET	Positive valence pictures	TAL
Kumari et al. 2003	MDD, CON, MDD vs. CON	44.00	6 (6/0)	47.00	6 (6/0)	Y (100 %)	Picture-caption pairs task	Positive P-C vs. Reference P-C	TAL
Johnston et al. 2015	MDD, CON, MDD vs. CON	50.79	21(15/6)	46.14	19(15/4)	Y (100 %)	Pessiglione task	Win trails	MNI
Mucci et al. 2015	SZ, CON, SZ vs. CON	31.91	22 (12/10)	33.10	28 (10/18)	Y (100 %)	MID	Reward vs. neutral	TAL
Choi et al. 2014	SZ vs. CON	29.10	17 (7/10)	29.10	15 (5/10)	Y (100 %)	EET	Hedonic vs. neutral condition	TAL
da Silva Alves et al. 2013	SZ vs. CON	34.55	12(0/12)	22.70	10 (0/12)	N	MID	Reward vs. no outcome	TAL
Esslinger et al. 2012	SZ vs. CON	27.10	27 (7/20)	27.80	27 (20/7)	N	Reward task	Reward vs. control condition	MNI
Nielsen et al. 2012b	SZ vs. CON	29.70	31 (9/22)	25.90	31 (9/22)	N	MID	Gain vs. no-gain	TAL
Grimm et al. 2012	SZ vs. CON	28.90	23 (17/6)	30.30	23 (17/6)	Y (100 %)	Reward task	Food vs. control stimuli	MNI
Morris et al. 2012	SZ, CON, SZ vs. CON	32.90	16 (8/8)	13.00	21 (7/14)	Y (100 %)	Reward-related task	Unexpected reward	MNI
Dowd and Barch 2012	SZ vs. CON	33.20	20 (6/14)	31.44	25 (7/18)	Y (100 %)	Reward prediction task	Money vs. no-money	TAL
Ursu et al. 2011	SZ, CON, SZ vs. CON	28.70	20 (7/13)	28.80	20 (5/15)	Y (73.91 %)	EET	Pleasant stimuli	TAL
Harvey et al. 2010	SZ, CON, SZ vs. CON	30.70	26 (13/13)	30.40	30 (11/19)	Y (80 %)	EET	Positive pictures vs. neutral pictures	TAL
Koch et al. 2010	SZ, CON, SZ vs. CON	29.70	20 (8/12)	35.20	19 (7/12)	Y (94.7 %)	RLT	Positive PE	TAL
Dowd and Barch 2010	SZ, CON, SZ vs. CON	36.25	32 (11/21)	36.80	40 (14/26)	Y (100 %)	EET	Positive vs. neutral	TAL
Simon et al. 2010	SZ vs. CON	25.20	15 (5/10)	26.30	15 (5/10)	Y (100 %)	MID	Reward vs. no reward	MNI
Waltz et al. 2010	SZ vs. CON	37.80	17 (4/13)	37.80	17 (5/12)	Y (100 %)	MID	Gain vs. loss	MNI
Waltz et al. 2009	SZ vs. CON	37.10	18 (4/14)	37.70	18 (5/13)	Y (100 %)	Pavlovian conditioning task	Positive TDE vs. negative TDE	TAL
Walter et al. 2009a	SZ, CON, SZ vs. CON	33.00	16 (9/7)	38.00	16 (8/8)	Y (100 %)	MID	Reward	MNI
Reske et al. 2007	SZ vs. CON	35.30	10	37.40	10 (4/6)	N	EET	Happiness	MNI
Schlagenhauf et al. 2009	SZ, CON, SZ vs. CON	30.10	15 (3/12)	30.10	15 (3/12)	N	MID	Gain vs. no outcome	MNI
Murray et al. 2008	SZ, CON, SZ vs. CON	26.00	12 (3/9)	26.00	13 (4/9)	Y (61.54 %)	RLT	Reward prediction error vs. neutral prediction error	MNI
Jensen et al. 2008	SZ vs. CON	36.50	13 (4/9)	37.60	13 (3/10)	Y (100 %)	RLT	CS +/ CS-	MNI
Schlagenhauf et al. 2008	SZ, CON, SZ vs. CON	31.80	10 (1/9)	30.50	10 (1/9)	Y (100 %)	MID	Gain vs. no money	MNI
Juckel et al. 2006a	SZ, CON, SZ vs. CON	30.60	10 (8/2)	31.50	10 (8/2)	Y (100 %)	MID	Gain vs. no outcome	TAL
Juckel et al. 2006b	SZ, CON, SZ vs. CON	31.70	10 (0/10)	26.80	10 (0/10)	N	MID	Gain vs. no outcome	TAL

*MID* = Monetary Incentive Delay Task; *EET* = Emotional Experience/regulation Task; *RLT* = Reward Learning Task; *WOF* = Wheel of Fortune task; *MNI* = Montreal Neurological Institute coordinates; *TAL* = Talairach and Tournoux coordinates

### Within-group ALE analysis

#### Healthy controls

**Fig. 2:**
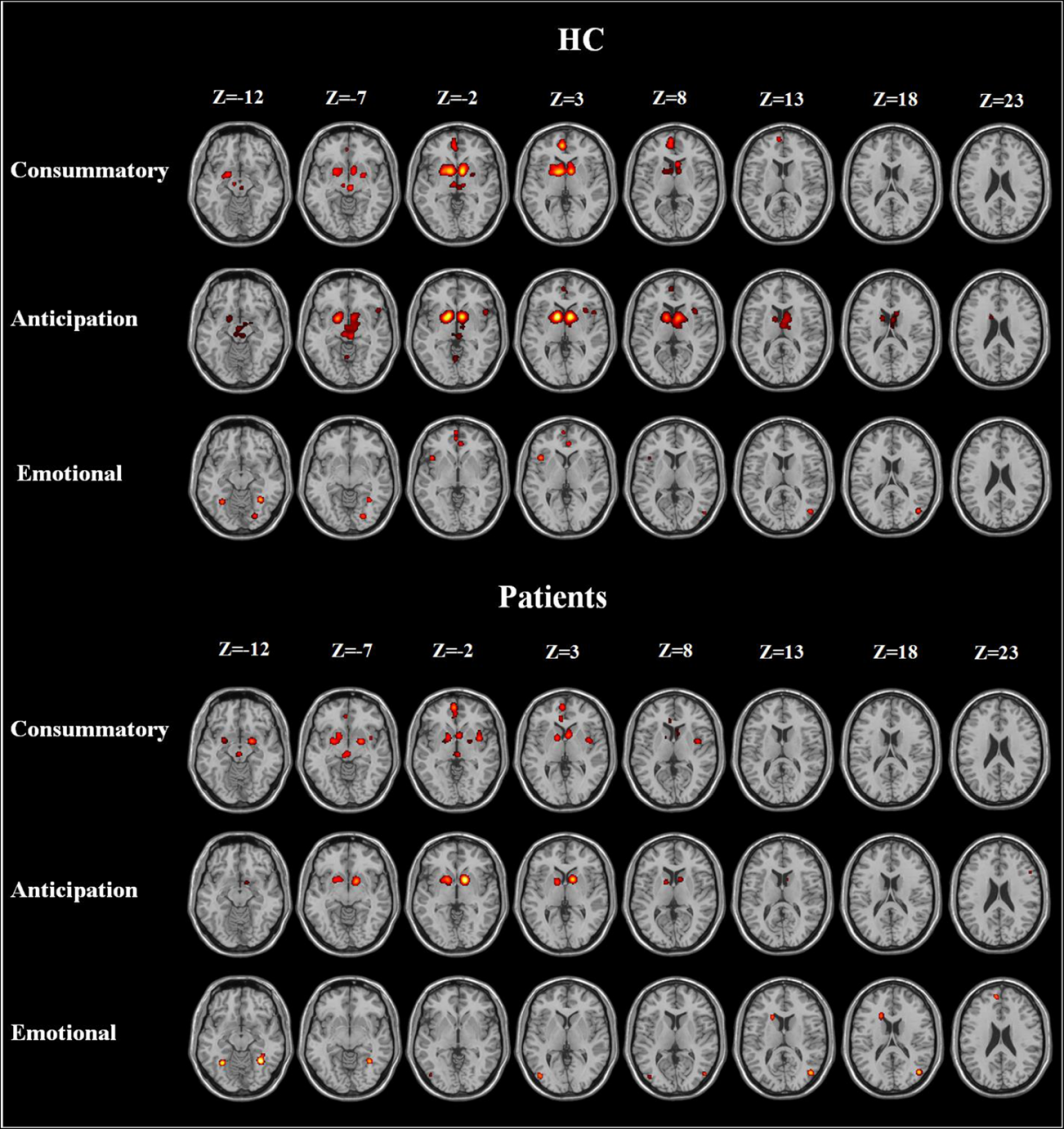
Significant ALE maps (FDR corrected, *p* < 0.01, cluster size >400 mm3) of within-group analysis in healthy control group (HC) and transdiagnostic patient group (mixed MDD and SZ) for reward consummatory, reward anticipation, and emotional processing

For reward consummatory (16 studies and 160 foci), ALE revealed a set of subcortical areas, including bilateral globus pallidus (GPe), bilateral red nucleus, right caudate body, left substania nigra (SN) and right putamen, left parahippocampal gyrus, as well as left medial frontal gyrus (mPFC). For reward anticipation (17 studies and 99 foci), ALE results showed robust activation of a broad cortical-subcortical network, including left GPe, right caudate head, left caudate body, bilateral red nucleus, right inferior frontal cortex (IFG), right superior temporal gyrus (STG), right insula, left mPFC and some midbrain areas, such as mammillary body. For the emotional processing (9 studies and 94 foci), several regions in frontal gyrus were activated to a greater degree, such as mPFC, MFG and IFG, as well as the middle temporal gyrus and fusiform gyrus (*p* < 0.01, FDR corrected, cluster size > 400 mm3, Table 2 and Fig. 2).

**Table 2:** Within-group ALE meta-analysis results in healthy controls and patients (mixed MDD and SZ)

Cluster	Anatomical region	BA	X	Y	Z	Volume mm^3^	Maximum ALE Value (10^−3^)
Healthy controls							
Reward consummatory							
1	R Lateral Globus Pallidus		10	6	−2	10384	25.07
	L Lateral Globus Pallidus		−10	4	0		24.63
	L Parahippocampal Gyrus	34	−18	0	−12		13.16
	R Caudate Body		8	18	6		12.73
2	L Medial Frontal Gyrus.		−4	48	2	2864	21.02
3	R Red Nucleus		4	−22	−8	1432	14.40
	L Red Nucleus		−4	−16	−4		9.87
	L Substania Nigra		−8	−16	−12		8.77
4	R Putamen		26	0	−6	440	10.84
Reward anticipation							
1	L Lateral Globus Pallidus		−12	6	0	20736	49.99
	R Caudate Head		10	6	0		46.31
	R Red Nucleus		6	−26	−8		19.19
	L Red Nucleus		−6	−22	−8		14.62
	L Caudate Body		−12	4	18		11.84
	R Subthalamic Nucleus		12	−12	−8		10.90
	L Mammillary Body		0	−12	−10		10.89
	R Mammillary Body		4	−12	−8		10.89
	R Hypothalamus		10	−6	−8		10.87
2	R Inferior Frontal Gyrus	47	50	14	−2	712	12.80
	R Superior Temporal Gyrus	38	52	18	−8		10.66
3	R Insula	13	36	16	6	552	11.67
4	L Medial Frontal Gyrus	10	−2	54	4	424	10.84
5	L Anterior Lobe.Culmen.		0	−60	−6	416	10.58
Emotional processing							
1	L Medial Frontal Gyrus	32	−8	12	46	1000	18.60
2	R Posterior Lobe.Declive		34	−52	−12	768	20.80
3	L Medial Frontal Gyrus	9	0	46	32	688	16.68
4	L Inferior Frontal Gyrus		−42	18	2	624	16.42
5	R Middle Temporal Gyrus	39	46	−72	14	528	16.92
6	R Fusiform Gyrus	19	24	−80	−10	560	16.04
7	R Inferior Frontal Gyrus	9	42	8	30	520	17.43
8	L Medial Frontal Gyrus	10	−2	60	−2	472	12.04
	L Medial Frontal Gyrus	10	−2	50	−4		10.96
9	L Posterior Lobe.Declive.		−30	−56	−14	456	17.93
10	R Cuneus	19	28	−84	32	496	17.23
11	R Anterior Cingulate	32	6	40	0	456	17.93
Patients (mixed MDD and SZ)							
Reward consummatory							
1	L Lateral Globus Pallidus		−12	4	0	2456	13.93
	L Parahippocampal Gyrus	34	−24	0	−10		13.69
	L Putamen		−14	8	−8		12.20
2	L Anterior Cingulate	10	−4	54	−2	1952	18.72
	L Anterior Cingulate	24	−6	38	2		9.12
3	R Insula	13	38	4	−4	1544	14.48
4	R Amydala		22	−2	−10	1424	16.94
5	R Caudate Head		6	8	0	1192	16.25
6	L Red Nucleus		0	−22	−8	1144	11.76
7	L Inferior Frontal Gyrus	9	−54	12	30	488	12.78
Reward anticipation							
1	R Putamen		14	8	−2	3168	29.48
2	L Putamen		−14	10	−4	2536	16.57
3	R Inferior Frontal Gyrus	9	54	20	26	528	13.44
4	L Superior Frontal Gyrus	9	−1	49	32	400	10.66
Emotional processing							
1	R Declive		34	−52	−12	1168	21.69
2	R Middle Temporal Gyrus	39	46	−72	14	816	17.43
3	R Inferior Frontal Gyrus	9	44	8	30	776	18.06
4	L Declive		−30	−56	−14	680	18.96
5	R Cuneus	19	29	−85	32	576	17.52
6	L Caudate Body		−18	20	14	496	11.39
7	L Middle Occipital Gyrus	19	−44	−78	2	432	11.38
8	L Superior Frontal Gyrus	9	−6	52	24	432	11.89

*p <* 0.01, FDR corrected, cluster size >400 mm^3^; *BA =* Brodmann area

#### Patients (mixed MDD and SZ)

For reward consummatory (11 studies and 68 foci), ALE analysis found seven statistically significant activation clusters, including left GPe, right caudate head, left red nucleus, right insula, left anterior cingulate gyrus (ACC), IFG and amydala. Reward anticipation (14 studies and 63 foci) was related to the significant activation of putamen, superior frontal gyrus (SFG) and IFG. As to the emotional processing (7 studies and 83 foci), increased likelihood of activation was observed in several frontal gyrus, temporal gyrus, occipital gyrus, as well as caudate body (*p* < 0.01, FDR corrected, cluster size > 400 mm3, Table 2 and Fig. 2). The results of within-group analysis results in MDD or SZ separately showed the similarly distribution and activation pattern (details see Table S1 and Fig. S1).

### Between-group ALE analysis in a transdiagnostic approach across MDD and SZ

#### Consummatory anhedonia

**Fig. 3:**
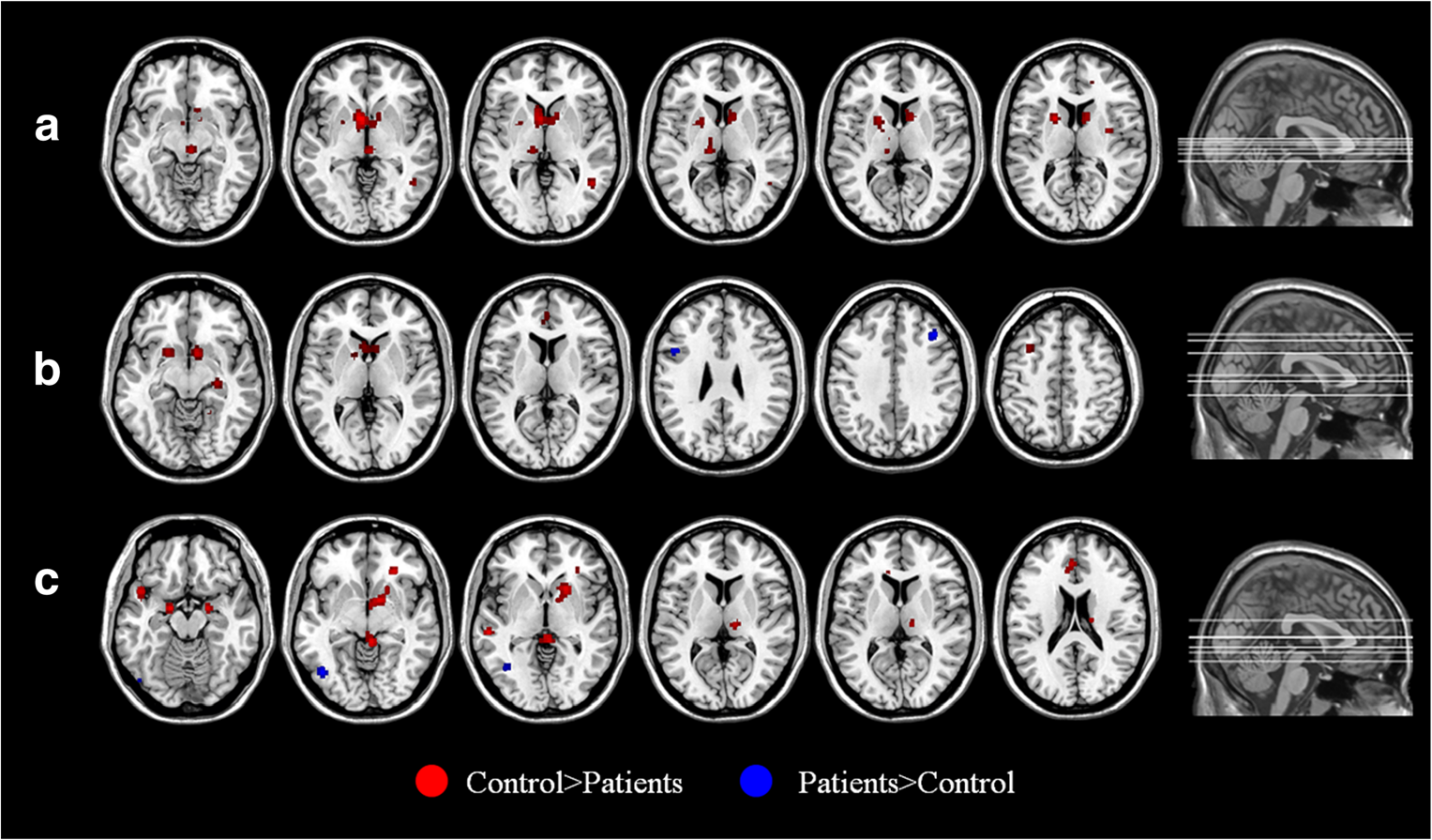
Significant ALE maps (FDR corrected, *p* < 0.01, cluster size >400 mm3) of between-group analysis in a transdiagnostic approach across MDD and SZ for three subdomains: (**a**) consummatory anhedonia (**b**) anticipatory anehdonia and (**c**) emotional processing. Red and blue areas depict regions with increased and decreased likelihood of activation in the healthy controls than that in patients, respectively

For consummatory anhedonia (29 studies and 151 foci), ALE analysis revealed five statistically significant clusters with decreased likelihood of activation in patients compared to controls (Fig. 3 and Table 3), including bilateral caudate head and body, bilateral GPe, left putamen, right red nucleus, left ventral lateral nucleus and pulvinar, as well as MTG (*p* < 0.01,, FDR corrected, cluster size > 400 mm^3^). No brain regions showed increased likelihood of activation in patients compared to controls.

**Table 3:** ALE results in a transdiagnostic approach across MDD and SZ

Cluster	Anatomical Region	BA	X	Y	Z	Volume mm^3^	Maximum ALE Value (10^−3^)
Consummatory anhedonia							
Control > patients							
1	L Caudate Head		−6	8	−2	4696	21.54
	R Caudate Body		12	10	12		14.07
	R Caudate Head		6	6	2		12.22
	R Lateral Globus Pallidus		14	6	−6		11.06
	R Caudate Body		10	16	−8		10.26
2	L Putamen		−20	8	12	1216	18.35
	L Putamen		−26	2	4		10.70
	L Putamen		−24	4	−4		9.51
	L Lateral Globus Pallidus		−18	−4	8		9.25
3	R Red Nucleus		4	−22	−6	800	18.22
4	L Pulvinar		−12	−24	4	608	14.71
	L Ventral Lateral Nucleus		−10	−12	6		9.73
5	R Middle Temporal Gyrus	37	46	−56	2	496	16.42
Anticipatory anhedonia							
Control > patients							
1	R Caudate Head		8	12	−4	5248	21.63
	L Putamen		−14	8	−2		21.38
	L Caudate Head		−4	14	2		10.62
2	R Hippocampus		28	−20	−8	560	13.83
3	L Anterior Cingulate	32	0	46	8	560	9.84
	L Medial Frontal Gyrus	10	−4	52	14		7.77
4	L Middle Frontal Gyrus	8	−32	14	52	488	11.88
5	R Parahippocampal Gyrus	19	22	−48	−4	464	10.95
Patients > control							
1	R Middle Frontal Gyrus	9	38	26	38	560	13.87
2	L Inferior Frontal Gyrus	9	−46	12	30	504	13.98
Emotional processing							
Control > patients							
1	R Lateral Globus Pallidus		14	4	−4	2904	17.11
	R Amygdala		20	−4	−12		15.81
	R Putamen		20	14	−2		15.73
2	R Anterior Lobe. Culmen		6	−38	−4	1640	17.06
	L Parahippocampal Gyrus	30	−6	−38	4		11.84
3	R Inferior Frontal Gyrus	47	26	32	−8	888	17.17
4	R Medial Dorsal Nucleus		12	−20	6	832	12.56
	R Ventral Lateral Nucleus		14	−16	14		11.42
5	L Medial Frontal Gyrus	9	−4	40	22	736	12.87
	L Anterior Cingulate	32	−10	32	12		9.50
6	L Superior Temporal Gyrus	38	−46	14	−12	688	15.57
7	L Amygdala		−18	−4	−14	448	16.22
8	L Middle Temporal Gyrus	22	−56	−28	0	400	15.16
Patients > control							
1	L Middle Occipital Gyrus	37	−44	−66	−6	1160	13.15
	L Fusiform Gyrus	19	−46	−74	−10		9.57

*p <* 0.01, FDR corrected, cluster size >400 mm^3^; *BA* = Brodmann area

#### Anticipatory anhedonia

For anticipatory anhedonia (30 studies and 119 foci), prefrontal cortex and striatal areas showed significantly different activity (Fig. 3 and Table 3). Decreased likelihood of activation was observed in bilateral caudate head, left putamen, right hippocampus and parahippocampus, ACC, mPFC and MFG (*p* < 0.01, FDR corrected, cluster size > 400 mm^3^). Increased likelihood of activation was observed in the left IFG and MFG.

#### Emotional processing

For the emotional experience task (31 studies and 163 foci), decreased likelihood of activation was observed in a number of brain areas from the cortical-subcortical network (Fig. 3 and Table 3), including right GPe, right putamen, right medial dorsal nucleus and ventrolateral nucleus of thalamus, bilateral amygdala, left parahippocampal gyrus, right IFG, left mPFC, left ACC and STG/MTG (*p* < 0.01, FDR corrected, cluster size > 400 mm^3^). Increased likelihood of activation was observed in the left middle occipital gyrus and fusiform gyrus.

### Between-group ALE analysis in MDD or SZ

#### MDD

**Fig. 4:**
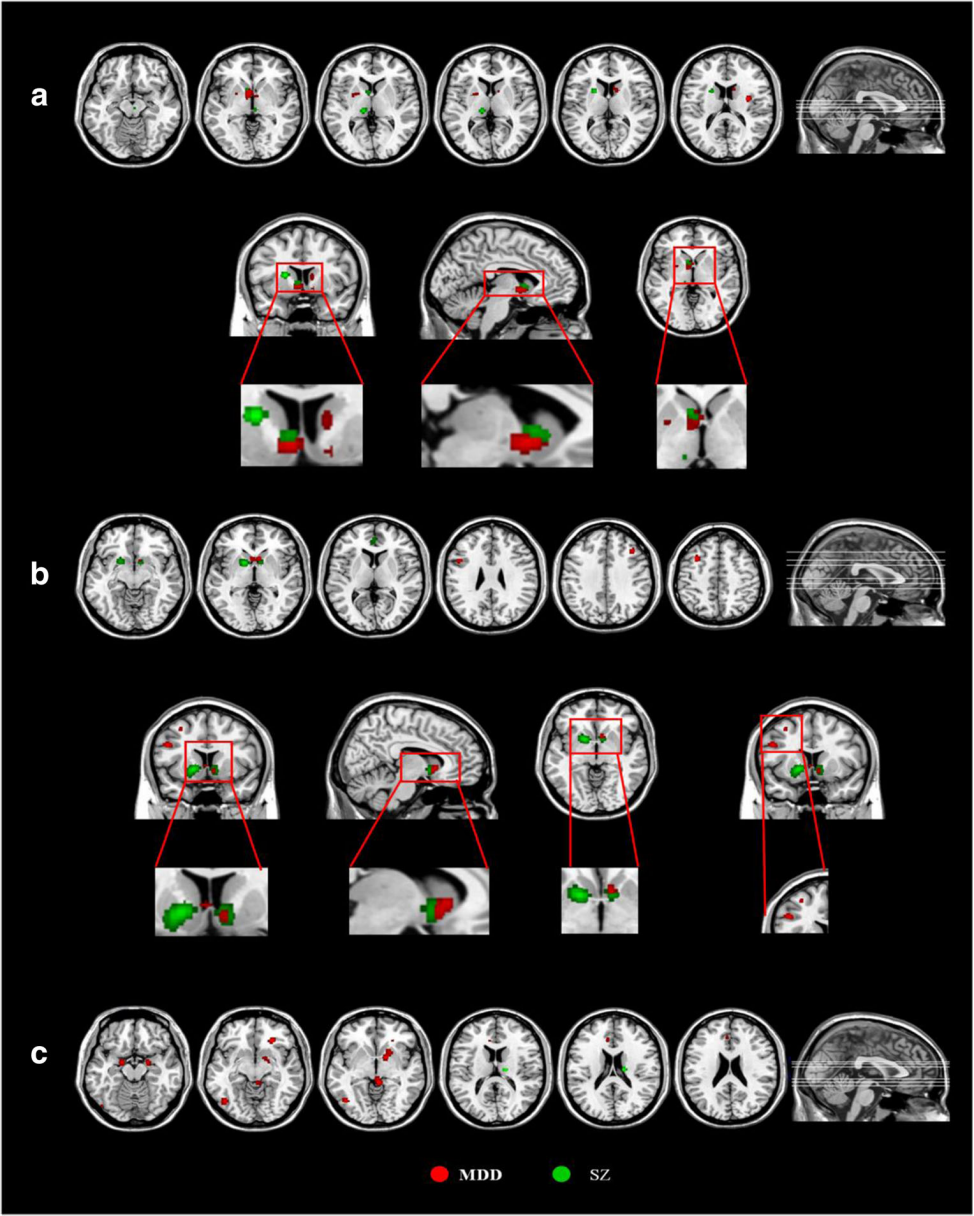
Significant ALE maps (FDR corrected, *p* < 0.01, cluster size >400 mm3) of between-group analysis in MDD or SZ respectively for three subdomains: (**a**) consummatory anehdonia (**b**) anticipatory anhedonia and (**S**) emotional processing. Red areas depict the regions with significant different activation between MDD patients and healthy controls. Green areas depict regions with significant different activation between SZ patients and healthy controls. ALE clusters are projected on a standard anatomical template in axial orientation, referring to Talairach space

For consummatory anhedonia in MDD (21 studies and 107 foci), decreased likelihood of activation was observed in the left GPe, right caudate body, left putamen, right insula and left ACC (*p* < 0.01, FDR corrected, cluster size > 400 mm3; Table S2 and Fig. 4). No areas with increased likelihood activation were observed.

For anticipatory anhedonia in MDD (17 studies and 89 foci), decreased likelihood of activation was observed in bilateral caudate head and left MFG, and increased activation was observed in left IFG and right MFG.

For emotional processing in MDD (25 studies and 124 foci), decreased likelihood of activation was observed in right GPe and putamen, bilateral amygdala and anterior lobe, right IFG and left ACC, and increased activation was observed in middle occipital gyrus and fusiform gyrus.

#### SZ

For consummatory anhedonia in SZ (8 studies and 44 foci), decreased likelihood of activation was observed in left putamen and caudate head, pulvinar and right red nucleus (*p* < 0.01, FDR corrected, cluster size > 400 mm3; Table S3 and Fig. 4). No areas with increased likelihood of activation were observed.

For anticipatory anhedonia in SZ (13 studies and 30 foci) decreased likelihood of activation was observed in left putamen, ACC, and mPFC and right caudate head. No areas with increased likelihood of activation were observed.

For emotional processing in SZ (6 studies and 39 foci), decreased likelihood of activation was observed in the right ventral lateral nucleus. No areas with increased likelihood of activation were observed.

## Discussion

This review used a transdiagnostic, meta-analytic approach (ALE) to explore the neuronal basis of anhedonia across three subdomains: consummatory anhedonia, anticipatory anhedonia and emotional processing. Results revealed that consummatory anhedonia was associated with decreased activation in ventral basal ganglia areas, ventral tegmental area (VTA) and various subcortical areas. Anticipatory anhedonia was related to dysfunctions within basal ganglia regions like caudate and putamen, as well as various subregions of prefrontal cortex, such as anterior cingulate, medial frontal gyrus and middle frontal gyrus. Emotional processing of positive-valence stimuli was related to more distributed dysfunction, involving more of basal ganglia (putamen and GPe), thalamus, limbic regions (amygdala and parahippocampal gyrus), prefrontal cortex (IFG, mPFC, ACC), STG and middle occipital gyrus. Meta-analyses of MDD and SZ respectively revealed similar impairments in brain regions and activation patterns in consummatory and anticipatory anhedonia in these two diseases, but differences in emotional processing.

### The neural correlates of different reward processing component

The within-group analysis results of healthy controls showed that the three reward processing components related with anhedonia were differentially recruited the subregions in the basal ganglia and forebrain. Specifically, the reward consummatory processing was mainly concentrated on a series of subcortical regions including GPe, ventral striatum, SN, as well as mPFC and extended orbitofrontal cortex, which is consistent with a number of animal and human studies (Der-Avakian and Markou 2012). Recent researches suggests that the opioid and GABA-ergic systems may play important role in that hedonic responses (at least to primary sensory stimuli). On the other side, reward anticipatory processing was more involved in dorsal striatum and cortical areas except for the ventral striatum and medial profrontal areas,, such as inferior frontal gyrus and insula. Those results highlighted importance of frontostriatal circuitry in reward anticipatory/prediction processing (Barch and Dowd 2010; Jarbo and Verstynen 2015) and indicated that further investigation are needed on identifying the connectivites between subareas of frontal cortex and striatum for specific reward component. Unlike the neural correlates of reward consummatory and anticipation, the within-group analyses for emotional processing task revealed much more widely distributed cortical areas in healthy controls, but not many subcortical regions. Those results suggested that the activated brain areas in emotional experience task may be the synthesis of various cognitive and emotional response related with reward. Furthermore, both the transdiagnostic analysis with mixed patients group and the analysis with MDD or SZ group separately showed a similarly contributed but less activated pattern with that of healthy controls in each of the subdomains of anhedonia.

### Transdiagnostic neural markers of anhedonia

In both MDD and SZ, anhedonia is considered to be a core clinical characteristic (Griffiths et al. 2014; Treadway et al. 2012; Wang et al. 2008). Animal studies have demonstrated parallel substrates across different disorders associated with altered anhedonia-related processing (Barr and Phillips 2002; Der-Avakian and Markou 2010). Therefore, researchers suggested that common pathological mechanisms could underlie anhedonia-related processing, supporting a transdiagnostic approach to reveal these underlying cognitive and neurobiological mechanisms. In this review, we applied an ALE meta-analysis to MDD and SZ to provide an integrated framework of the neural bases of anhedonia.

Based on the results of this transdiagnostic meta-analysis, consummatory anhedonia is related to decreased activation in several subcortical regions, such as caudate head and body, putamen, red nucleus, GPe, and pulvinar. Animal studies have demonstrated involvement of the ventral striatum and ventral pallidum in the experience of pleasure and hedonic perception of rewards (Berridge and Kringelbach 2008; Kelley et al. 2002). Furthermore, patients with ventral pallidus damage have significantly reduced responses to rewards or pleasant stimuli (Miller et al. 2006; Vijayaraghavan et al. 2008). Consistent with previous findings, our meta-analysis further implicated the opioid and GABA-ergic systems in the nucleus accumbens shell and its projections to the ventral pallidum, in deficits of in-the-moment hedonic experience. Specifically, reduced caudate activation has been reported to be associated with blunted processing of incentive perception and reinforcement learning in both MDD and SZ (Dowd and Barch 2010; Pizzagalli 2014; Pizzagalli et al. 2009). A more recent conceptualization of pulvinar function posited that this region plays an important role in emotional salience and awareness (Hamilton et al. 2012; Pessoa and Adolphs 2010) and has strong bidirectional connectivity with insula and dACC (Mufson and Mesulam 1984). Given this role of the pulvinar, we propose that low-level pulvinar activation may blunt hedonic experience through insufficient attention and awareness of reward-related information. To summarize, ALE meta-analysis on consummatory anhedonia processing emphasized the critical role of ventral basal ganglia and medial prefrontal pathways in generating the in-the-moment hedonic value representation and experience.

For anticipatory anhedonia, ALE showed involvement of the caudate and putamen, as well as several prefrontal subregions, such as dACC (BA32), mPFC and MFG. Previous studies suggested that multiple prefrontal-striatal pathways and reciprocal connections within subregions of PFC play an important role in regulating behavioral response to rewards or pleasurable stimuli in the motivational/anticipatory stage (Der-Avakian and Markou 2012; Salamone and Correa 2012). For instance, disruption of glutamatergic signaling between the mPFC and nucleus accumbens resulted in avolition for rewards (Faure et al. 2010). Moreover, striatal lesions or dopamine depletion in striatum or dACC impaired the computation of effort-related costs, which then lead to a deficit of effort-based decision-making as the expected rewards were discounted (Croxson et al. 2009). Preclinical studies further stressed that dACC plays an important role in signaling the net value (benefits minus costs) during social interactions (Apps and Ramnani 2014). In brief, during the anticipation stage for reward or pleasurable stimuli, abnormal activation in mPFC, dACC and MFG may lead to failure to anticipate forthcoming rewards, including updating/maintaining the value of pleasant stimuli, effort-value computation, decision-making to engage in goal-directed activity and monitoring incentive-based behavioral responses (Haber and Knutson 2010; Pujara and Koenigs 2014; Wallis and Kennerley 2010). The results of our meta-analysis consistently indicated that a number of brain regions and pathways, involving many neurotransmitter systems, mediated different aspects of anhedonia-related deficits. Consequently, this preliminary neural framework supports the conceptualization of anhedonia as a symptom with multiple components. Rigorous experimental paradigms and careful designs should be carried out in future work to provide a more refined description of anhedonia and to separate the neural bases of each aspect of anhedonia-related processing.

Our ALE analysis for anticipatory anhedonia also revealed reduced activation of the hippocampus and parahippocampal gyrus, which could impair retrieval of previous autobiographical memories and their incorporation into working memory to represent the value of a reward and anticipate a pleasurable stimulus (Adcock et al. 2006; Zhu et al. 2012; Mazgaj et al. 2015). However, we did not observe any significant group difference between patients and controls in orbitofrontal cortex (OFC), which is considered to be a key cortical region involved in anhedonia, in particular in the reward value presentation (Kringelbach 2005; Pizzagalli 2014). One possible explanation is that detection of OFC is challenging for functional MRI experiments using regular EPI sequences due to strong magnetic susceptibility variation near the air-filled sinuses and corresponding signal loss and distortion (Faro et al. 2006; Osterbauer et al. 2006). Future investigation of this unique structure will benefit from advances in imaging sequences or MRI hardware.

Given the complex neural pathways in the frontal-striatal-thalamic circuitry, it is necessary to further divide each subcortical structure (e.g., striatum) into subregions to investigate the function of different cortical-subcortical connections. For instance, the striatum can be divided into three functional subregions (i.e., limbic, associative and sensorimotor) based on afferent inputs from the frontal cortex (Haber 2010; Haber et al. 2000; Iversen 2010; van den Bos et al. 2014). Consummatory anhedonia is mainly associated with limbic/ventral striatum, including anterior and ventral caudate and putamen (Corral-Frias et al. 2015; Pizzagalli et al. 2009), whereas anticipatory anhedonia is associated with both limbic/ventral striatum and associative striatum, including the dorsal caudate and anterior part of the dorsal putamen (Delgado 2007; Dichter et al. 2009; Mucci et al. 2015). The results of our meta-analysis provide new evidence that dysfunction in distinct frontal-striatal pathways contributes to the different components of anhedonia-related processing. Based on this preliminary neural framework, further studies are needed to identify the functional and structural connections underlying these components of anhedonia-related processing.

ALE analysis of the emotional experience task revealed more widely distributed differences between patients and controls. In addition to blunted activation in basal ganglia and thalamus, a number of regions in limbic areas, prefrontal cortex, temporal cortex, and cerebellum also showed decreased activation in patients, whereas the visual association cortices showed increased activation. Given that the emotional experience task required no response and may include various cognitive and affective components, it is unclear whether those different activations reflect only the neural bases of anhedonia. Recent resting-state fMRI showed that different neural circuits were engaged in the emotional task-general and emotion task-specific processing (Cole et al. 2014), which would be differently activated by the emotional experience task between controls and patients with affective symptoms. Moreover, functional connectivity analysis demonstrated distinct circuits associated with severity of general affective symptoms (i.e., depression) and anhedonia (Gabbay et al. 2013). Indeed, a set of brain regions, including the anterior and posterior cingulate, mPFC, basal ganglia and visual areas, were activated during passive viewing of positive-valence stimuli, but the ventral striatum and mPFC were the key regions correlated with self-reported anhedonia severity (Epstein et al. 2006; Harvey et al. 2010). Therefore, more elaborate experimental designs will be required to explore the underlying neural bases of general mood-related processing and specific emotional experiences (e.g., hedonia).

### Disease-specific findings: MDD and SZ

To further explore anhedonia-related neural mechanisms and identify potential disease-specific or task-specific confounders, ALE analysis was compared between MDD and SZ patients across the three anhedonia subdomains. In general, anhedonia-related impairment was consistent between MDD and SZ patients across the consummatory and anticipatory stages. Those findings suggest that the neural substrates involved in accurate appraisal of positive stimuli and generation of reward responses, but not the substrates underlying hedonic emotion arousal, are likely to be the core brain areas associated with anhedonia. Furthermore, these data provide a partial explanation for the discordance between self-reported trait pleasure and momentary pleasure in previous behavioral studies: MDD and SZ patients reported normative affective ratings in response to evocative laboratory stimuli, but low positive affect and pleasurable experiences on evaluation of inventories (Osuch et al. 2009; Pizzagalli 2010; Strauss 2013; Treadway and Zald 2011). From a cognitive processing pespective, laboratory-based assessment of consummatory anhedonia may reflect the capacity for hedonic experience, while the patient self-reports in clinical interviews and inventories might reflect both the hedonic experience deficit and the inability to accurately represent incentive experiences. Based on this interpretation, the theoretical definition of anhedonia for research and targeted treatment may need to be modified.

In contrast to the consummatory and anticipatory anhedonia domains, which were similar across MDD and SZ, emotional experience tasks showed striking differences. In MDD patients, decreased activation was much more widely distributed across brain regions, including basal ganglia, amygdala, frontal gyrus and cerebellum; SZ patients only showed decreased activation in thalamus. As previously noted, the emotional experience task requires no response and may reflect other cognitive and affective components. Our results support that the emotional experience task induced both affective/mood-related and anhedonia-specific responses. It is worth noting that only blunted activation in part of ventral basal ganglia was correlated with anhedonia in MDD patients viewing positive valence pictures (Epstein et al. 2006; Mitterschiffthaler et al. 2003; Osuch et al. 2009), which was highly consistent with the correlations between dysfunctional BOLD signals and anhedonia in a consummatory anhedonia task (Gradin et al. 2011; Pizzagalli et al. 2009). Overall, it appears that an emotional experience task, such as passively viewing pictures or words, may not be a pure measure of anhedonia (i.e., consummatory anhedonia) and may capture the affective responses related with general mood, which may influence results in populations with prominent depressive symptoms.

### Limitations

Potential limitations of this study should be noted. First of all, ALE technique does have its own limitations. Unlike the meta-analyses in which the complete activation maps are included, the data used in ALE were based solely on reported peak activation coordinates. Thereby ALE could not take into account those studies without any significant clusters reported, which may resulting in a systematic overestimation bias of the results. Besides, the present ALE software cannot conduct the correlation analysis or deal with covariates, thus the only way to consider the covariates was run follow-up analyses, e.g., only on experiments investigating only female subjects or unmedicated patients when considering the gender or medication effect. An additional limitation of the ALE is that it may be unable to assess subtle methodological differences in individual studies such as the thresholding used in the original studies, relative strength of activations/differences between groups and differences in preprocessing steps (Radua and Mataix-Cols 2012). Nevertheless, previous studies suggested that no individual study could significantly bias the results of ALE meta-analyses after including the sample size and number of reported foci into ALE algorithm, changing from fixed-effects to random-effects inference, and revising for multiple comparison correction (Eickhoff et al. 2009; Turkeltaub et al. 2012; Eickhoff et al. 2012; Kollndorfer et al. 2013). In the present study, the robustness of results was further supported by the reasonably coherent findings in within-group ALE analysis of various reward processing components and between-group analyses of subdomains of anhedonia, which were also consistent with previous neurobioglogical studies on animals.

Secondly, given that the patients in 31/35 MDD studies and 22/25 SZ studies were treated in their lifetime by antidepressant or antipsychotics, the drug effects on the brain activated pattern in reward-related processing are potential confounding factors which need to be addressed (Clarke et al. 2014; Boccia et al. 2015). Most antidepressant treatments act on the serotonergic or noradrenergic circuits, but not directly enhance Dopamine (DA) neurotransmission, which plays a significant role in motivation and reward processing (Berridge 2007; Treadway and Zald 2011). However, recent literature showed that the antidepressant mechanism such as increasing serotonin (5-HT) originating from midbrain raphe nuclei (RN), could sequentially decrease the dopamine inhibition and increased ventral striatal activity to reward in humans and animals (Dremencov et al. 2005; Dunlop and Nemeroff 2007; Ossewaarde et al. 2011; El Yacoubi et al. 2011). As for the antipsychotic treatment that directly impacts the DA neurotransmission, evidence showed that the first generation (typical) and second generation (atypical) antipsychotic medications had different effects on the neural correlates of reward/motivation tasks (Juckel et al. 2006a, b; Kirsch et al. 2007; Schlagenhauf et al. 2008). For instance, Kirsch et al. found that patients with typical antipsychotics showed reduced ventral striatal activation, while atypically treated patients showed significantly stronger activation of the right ventral striatum. Those results were further supported by a controlled, longitudinal study (Nielsen et al. 2012a) . The different impact of typical and atypical antipsychotics on reinforcement learning of reward processing were also supported by both behavioral evidences (Beninger et al. 2003; Keri et al. 2005) and neuroimaging data (Insel et al. 2014). Further clinical and basic studies are needed to reveal the underlying mechanism of those effects.

### Conclusion and future directions

In summary, our ALE meta-analysis supported characterization of anhedonia by alterations in reward processing, which contain multiple complicated components and rely on many brain regions within frontal-striatal circuitry. Specifically, consummatory anhedonia was associated with decreased activation in ventral striatum and pallidum, while anticipatory anhedonia was more associated with more substrates in frontal-striatal networks except the ventral striatum, which included the dorsal anterior cingulate, middle frontal gyrus and medial frontal gyrus. However, the emotional experience task (passive viewing of positive pictures or words) revealed mixed findings, including dysfunction in both the anhedonia-related and affective/mood processing regions. Therefore, the transdiagnostic approach holds promise for providing both overall and specific neurobiological frameworks of anhedonia.

Although our meta-analysis presents novel, meaningful evidence regarding the neurobiology of anhedonia, it only distinguished the neural substrates of motivation to pursue rewards and hedonic response to rewards. Future work is needed to dissect the different neurobiological pathways that are related to the various reward-processing subcomponents, such as perceiving pleasure, encoding reward value, calculating costs and benefits, learning from prior reinforcement, making decisions and execute the action to pursue a reward. It is important to note that anhedonia also plays an important role in several other psychiatric and neurological disorders, such as Parkinson’s disease (Loas et al. 2012), post-traumatic stress disorder (Frewen et al. 2012) and drug addiction (Balodis and Potenza 2015; Hatzigiakoumis et al. 2011), albeit in heterogeneous ways. Therefore, combining more categories of disorders and using a greater sample size in future studies should further elucidate the neural foundations of anhedonia.

## Electronic supplementary material

Below is the link to the electronic supplementary material.

ESM 1 (DOCX 2258 kb)
