# Supplementary material for: Mapping anhedonia-specific dysfunction in a transdiagnostic approach: an ALE meta-analysis
Source: Brain Imaging Behav. 2015 Oct 20;10(3):920–39. doi: 10.1007/s11682-015-9457-6 (PMC4838562; doi:10.1007/s11682-015-9457-6)
Supplement: Supplementary file 1 — (DOCX 2258 kb) [file 11682_2015_9457_MOESM1_ESM.docx]

**Supplemental Material**

**Mapping anhedonia-specific dysfunction in a transdiagnostic**

**approach: An ALE meta-analysis**

Bei Zhang, Pan Lin, Huqing Shi, Dost Öngür, Randy P Auerbach, Xiaosheng Wang, Shuqiao Yao, Xiang Wang

**Results**

**Within-group ALE analysis of MDD**

For reward consummatory (5 studies and 44 foci), MDD was related to right GPe, parahippocampal gyrus, insula, ACC and IFG, while bilateral putamen, left SFG and MFG were involved in reward anticipation (7 studies and 37 foci). And emotional processing (4 studies and 30 foci) was related to left caudate body, amygdale, posterior cingulate and STG (p < 0.01, FDR corrected, cluster size > 400 mm3 , Table S1 and Fig. S1).

**Within-group ALE analysis of SZ**

For reward consummatory of SZ (6 studies and 24 foci), left GPe, left putamen, right caudate head were activated to some degree. Bilateral GPe, left IFG and right caudate body was involved with reward anticipation (7 studies and 26 foci). As to emotional experience tasks (3 studies and 53 foci), activation areas were very similar in qualitative pattern to that of controls and MDD, that is related to a range of cortex regions, such as MFG (p < 0.01, FDR corrected, cluster size > 400 mm^3^ , Table S1 and Fig. S1).

**Table S1** Within-group ALE analysis results in MDD and SZ groups.

| **Major Depressive Disorder** | | | | | | | | | | |  | **Schizophrenia** | | | | | | | | |
| --- | --- | --- | --- | --- | --- | --- | --- | --- | --- | --- | --- | --- | --- | --- | --- | --- | --- | --- | --- | --- |
| **Cluster** | | **Anatomical Region** | **BA** | **X** | | **Y** | **Z** | | **Volume mm^3^** | **Maximum ALE Value** |  | **Cluster** | | **Anatomical Region** | **BA** | **X** | **Y** | **Z** | **Volume mm^3^** | **Maximum ALE Value** |
| **Reward consummatory** | | | | | | | | | | |  | **Reward consummatory** | | | | | | | | |
| **1** | L Anterior Cingulate | | 10 | -4 | 54 | | | -2 | 1376 | 0.018714 |  | **1** | L Lateral Globus Pallidus | |  | -12 | 4 | 0 | 1360 | 0.013751 |
| **2** | R Lateral Globus Pallidus | |  | 22 | -2 | | | -8 | 1008 | 0.014798 |  |  | L Putamen | |  | -14 | 8 | -8 |  | 0.012 |
| **3** | L Parahippocampal Gyrus | | 34 | -24 | 0 | | | -10 | 1008 | 0.013633 |  | **2** | R Caudate Head | |  | 6 | 8 | 0 | 976 | 0.016037 |
| **4** | R Insula | | 13 | 38 | 4 | | | -4 | 784 | 0.014047 |  |  |  | |  |  |  |  |  |  |
|  | R Insula | | 13 | 46 | -2 | | | 4 |  | 0.008157 |  |  |  | |  |  |  |  |  |  |
| **5** | L Inferior Frontal Gyrus | | 9 | -54 | 12 | | | 30 | 568 | 0.012775 |  |  |  | |  |  |  |  |  |  |
| **Reward anticipation** | | | | | | | | | | |  | **Reward anticipation** | | | | | | | | |
| **1** | R Putamen | |  | 16 | 10 | | | -2 | 1512 | 0.019814 |  | **1** | R Lateral Globus Pallidus | |  | 14 | 4 | -4 | 1672 | 0.015923 |
| **2** | L Putamen | |  | -22 | 8 | | | -6 | 712 | 0.0122 |  |  | R Caudate Body | |  | 10 | 8 | 10 |  | 0.008143 |
|  | L Sub-lobar. | |  | -12 | 8 | | | -8 |  | 0.007839 |  | **2** | L Lateral Globus Pallidus | |  | -12 | 4 | 0 | 1080 | 0.013258 |
| **3** | L Superior Frontal Gyrus | | 9 | -1 | 49 | | | 32 | 464 | 0.010661 |  | **3** | L Inferior Frontal Gyrus | | 45 | -42 | 20 | 14 | 368 | 0.008839 |
| **4** | R Middle Frontal Gyrus | | 6 | 24 | -8 | | | 62 | 416 | 0.009427 |  |  | L Inferior Frontal Gyrus | | 9 | -44 | 12 | 20 |  | 0.008602 |
| **Emotional processing** | | | | | | | | | | |  | **Emotional processing** | | | | | | | | |
| **1** | L Superior Temporal Gyrus | | 13 | -52 | -40 | | | 18 | 760 | 0.008681 |  | **1** | R Posterior Lobe.Declive | |  | 34 | -52 | -12 | 1000 | 0.021689 |
| **2** | L Caudate Body | |  | -16 | 24 | | | 18 | 656 | 0.007768 |  | **2** | R Middle Temporal Gyrus | | 39 | 46 | -72 | 14 | 624 | 0.017429 |
| **3** | L Posterior Cingulate | | 23 | -4 | -30 | | | 26 | 448 | 0.005967 |  | **3** | R Inferior Frontal Gyrus | | 9 | 44 | 8 | 30 | 648 | 0.018059 |
|  | R Cingulate Gyrus | | 23 | 2 | -26 | | | 30 |  | 0.005943 |  | **4** | L Posterior Lobe.Declive | |  | -30 | -56 | -14 | 496 | 0.018484 |
| **4** | L Amygdala | |  | -18 | -2 | | | -10 | 416 | 0.009324 |  | **5** | R Occipital Lobe.Cuneus | | 19 | 29 | -85 | 32 | 512 | 0.017521 |

Note: *p* < 0.01, FDR corrected, cluster size > 400 mm^3^; BA= Brodmann area.

**Table S2** Between-group ALE analysis results in MDD.

| **cluster** | **Anatomical Region** | **BA** | **X** | **Y** | **Z** | **Volume mm^3^** | **Maximum ALE Value** |
| --- | --- | --- | --- | --- | --- | --- | --- |
| **Consummatory anhedonia** | | | | | | | |
| **Control > MDD** | | | | | | | |
| 1 | L Lateral Globus Pallidus |  | 14 | 6 | -6 | 1552 | 0.010479 |
|  | L Anterior Cingulate | 25 | -4 | 4 | -4 |  | 0.016129 |
| **2** | R Insula | 13 | 34 | -4 | 16 | 520 | 0.018015 |
| **3** | R Caudate Body |  | 12 | 12 | 14 | 512 | 0.012189 |
| **4** | L Putamen |  | -26 | 2 | 4 | 448 | 0.010485 |
|  | L Putamen |  | -20 | 4 | 6 |  | 0.00982 |
|  | L Putamen |  | -24 | 4 | -4 |  | 0.009506 |
| **Anticipatory anhedonia** | | | | | | | |
| **Control > MDD** | | | | | | | |
| **1** | R Caudate Head |  | -4 | 16 | 2 | 1080 | 0.011712 |
|  | L Caudate Head |  | 8 | 14 | -2 |  | 0.011212 |
| **2** | L Middle Frontal Gyrus | 8 | -32 | 14 | 52 | 520 | 0.01356 |
| **MDD > Control** | | | | | | | |
| **1** | R Middle Frontal Gyrus | 9 | 38 | 26 | 38 | 560 | 0.013865 |
| **2** | L Inferior Frontal Gyrus | 9 | -46 | 12 | 30 | 504 | 0.013980 |
| **Emotional processing** | | | | | | | |
| **Control > MDD** | | | | | | | |
| **1** | R Amygdala |  | 20 | -4 | -12 | 2080 | 0.015801 |
|  | R Lateral Globus Pallidus |  | 14 | 4 | -4 |  | 0.015528 |
|  | R Putamen |  | 20 | 12 | -2 |  | 0.013768 |
| **2** | R Anterior Lobe. Culmen |  | 6 | -38 | -4 | 1376 | 0.017054 |
|  | L Anterior Lobe. Culmen |  | -4 | -40 | 4 |  | 0.009802 |
| **3** | R Inferior Frontal Gyrus | 47 | 26 | 32 | -8 | 680 | 0.016582 |
| **4** | L Amygdala |  | -18 | -4 | -14 | 488 | 0.016215 |
| **5** | L Anterior Cingulate | 32 | -8 | 32 | 20 | 400 | 0.009906 |
|  |  | 32 | -10 | 32 | 12 |  | 0.00949 |
|  |  | 32 | -4 | 38 | 24 |  | 0.00936 |
| **MDD > Control** | | | | | | | |
| **1** | L Inferior Occipital Gyrus | 19 | -44 | -68 | -6 | 880 | 0.013016 |
|  | L Fusiform Gyrus | 19 | -46 | -74 | -10 |  | 0.009572 |

Note: *p* < 0.01, FDR corrected, cluster size > 400 mm^3^; BA= Brodmann area.

**Table S3** Between-group ALE analysis results in SZ.

| **cluster** | **Anatomical Region** | **BA** | **X** | **Y** | **Z** | **Volume mm^3^** | **Maximum ALE Value** |
| --- | --- | --- | --- | --- | --- | --- | --- |
| **Consummatory anhedonia** | | | | | | | |
| **Control > SZ** | | | | | | | |
| **1** | R Red Nucleus |  | 4 | -22 | -6 | 696 | 0.015298 |
|  | R Red Nucleus |  | 2 | -18 | -14 |  | 0.009074 |
| **2** | L Putamen |  | -22 | 10 | 12 | 592 | 0.016482 |
| **3** | L Pulvinar |  | -12 | -24 | 4 | 520 | 0.014517 |
| **4** | L Caudate Head |  | -6 | 8 | 2 | 400 | 0.010413 |
|  | L Caudate Head |  | -8 | 12 | 0 |  | 0.010219 |
| **Anticipatory anheodnia** | | | | | | | |
| **Control > SZ** | | | | | | | |
| **1** | L Putamen |  | -14 | 8 | -2 | 2408 | 0.021325 |
| **2** | R Caudate Head |  | 10 | 10 | -4 | 1048 | 0.013213 |
| **3** | L Anterior Cingulate | 32 | 0 | 46 | 8 | 640 | 0.00984 |
|  | L Medial Frontal Gyrus | 10 | -4 | 52 | 14 |  | 0.007773 |
| **Emotional processing** | | | | | | | |
| **Control > SZ** | | | | | | | |
| **1** | R Ventral Lateral Nucleus |  | 16 | -16 | 18 | 448 | 0.011201 |

Note: *p* < 0.01, FDR corrected, cluster size > 400 mm^3^; BA= Brodmann area.


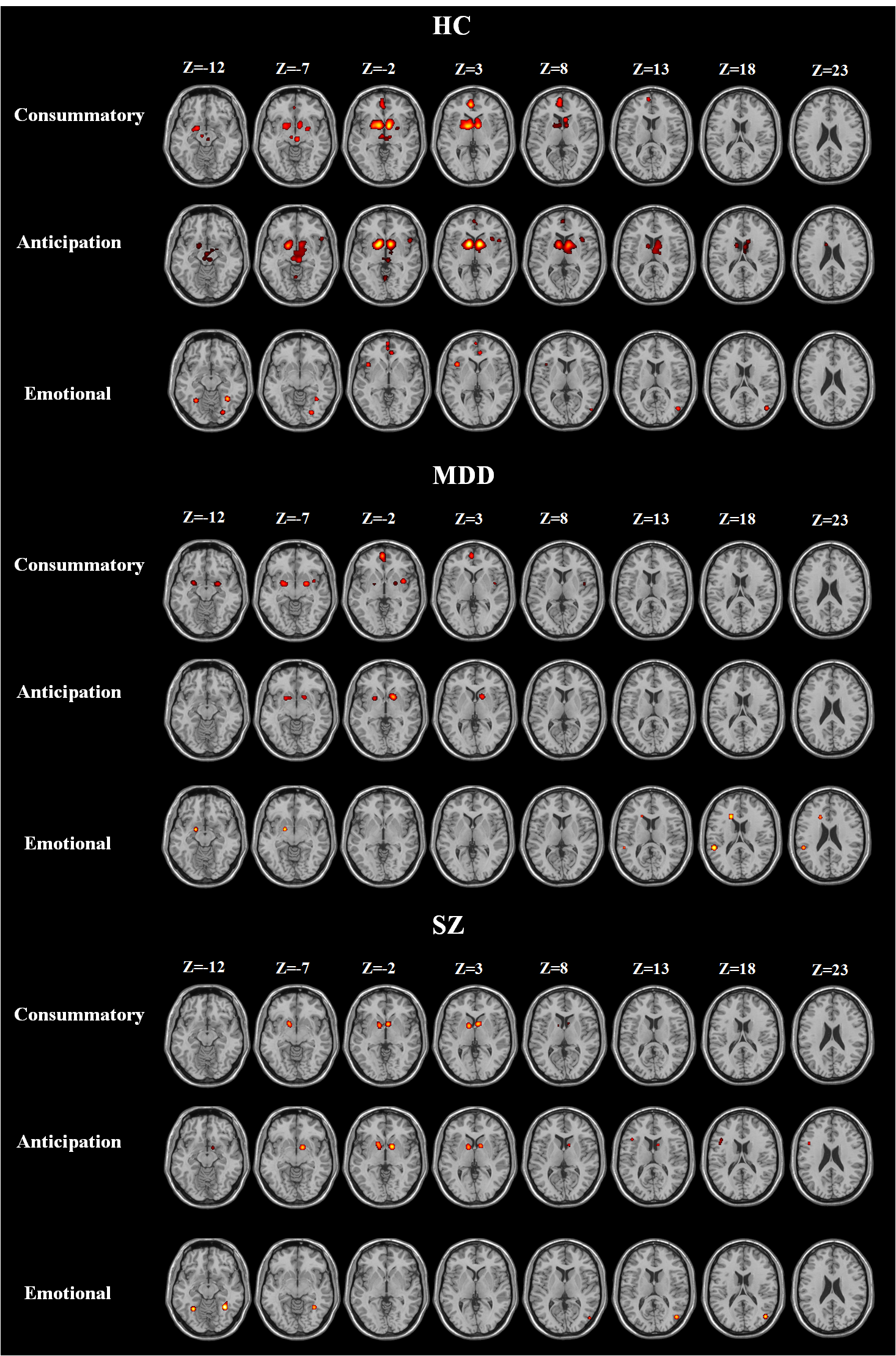


**Fig. S1** Significant ALE maps (FDR corrected, p < 0.01, cluster size >400 mm^3^) of within-group analysis in HC, MDD and SZ group for reward consummatory, reward anticipation and emotional processing.
